# An Investigation on the Preconditions and Diagnosis Methods for Alien Hand Syndrome

**DOI:** 10.7759/cureus.22381

**Published:** 2022-02-19

**Authors:** Aakash Pradhan, Akshay J Reddy, Avanthika Rajendran, Neel Nawathey, Mark Bachir, Hetal Brahmbhatt

**Affiliations:** 1 Health Sciences, California Northstate University, Rancho Cordova, USA; 2 Opthalmology, California Northstate University College of Medicine, Elk Grove, USA; 3 Health Sciences, Reed College, Portland, USA; 4 Psychiatry, Mercy General Hospital, Sacramento, USA

**Keywords:** ct scan, pet scans, dtt, mri images, alien hand syndrome

## Abstract

Although it is not a very common condition, people who have suffered from neuro-damage or neuro-diseases are at risk for developing a condition known as Alien hand syndrome (AHS). Individuals who have this condition are unable to control the movement of their hands for certain brief intervals of time. In order to improve upon the treatment of individuals with AHS, it is important that signs and symptoms of the disease are identified as soon as possible. The purpose of this investigation is to catalog the data regarding the pre-existing conditions and the method of diagnosis for AHS. Within the review, it was revealed that stroke was the most common pre-existing condition for the disease. Therefore, physicians who have stroke patients within their care should carefully monitor their condition in case they do develop AHS. Additionally, it was found that using an MRI machine was the most common method of diagnosing a patient with AHS. This was most likely because MRI scans provide the most information about a patient’s brain functionality which can be used to deduce if an individual has AHS.

## Introduction and background

Alien hand syndrome (AHS) is a rare clinical condition that is autonomic, unruly, and pointless in terms of upper limb movements. AHS is commonly thought to be a form of interhemispheric disconnection [1]. This condition is caused by several lesions in the supplementary motor area, corpus callosum, medial frontal lobe, posterior parietal, or thalamus. AHS most commonly appears in patients who have corticobasal degeneration, in fact over 60% of patients who have corticobasal degeneration also have AHS [2]. When patients with AHS touch their upper limb without visual direction, they lose control. Such types of motion do not have a certain objective and can be distinguished from choreic and athetotic movements [3]. Additionally, it is possible that they might experience a complicated sensation of limb unfamiliarity. This is different from phantom limb syndrome as this condition does not involve amputated patients feeling the presence of a nonexistent limb. The movements of AHS patients might be exceedingly strange at times that is difficult to tell if they are real or not, erroneously assumed to be functional. AHS can be caused by a variety of phenomena. Although stroke is one of the main causes of the disease, the illness is not linked to a specific vascular stroke syndrome [4]. Following neurosurgical procedures, AHS can still be present within patients. Other neurological abnormalities such as diminished motor spontaneity, speech hesitancy, apraxia, tactile dysnomia, frontal lobe, and dysfunction-related behaviors are also known to be linked to AHS [5]. Patients with AHS typically have ego-dystonic or unfavorable feelings about the infected limb. These symptoms will usually last for a long period of time, and the prognosis for recovery is dismal. In fact, over the past 20 years, there have only been seven instances recorded on PubMed where AHS patients had marginal recovery with mixed results, other studies have reported that AHS patients experienced little no to recovery [6-19]. Therapies aimed at muscle control, for example, botulinum toxin and neuromuscular blocking drugs, have proved beneficial in treating AHS [6]. With moderate efficacy, cognitive treatment approaches were applied with the cooperation of most patients.

## Review

AHS is an arduous disease to treat as it is one of the few neurological conditions that is characterized by a complete loss of control of one limb [1]. The defining characteristic of AHS is that the arm moves abruptly, and resembles the jerking movements of epilepsy. The challenge of diagnosing AHS is that though it is a neurological disease, there is not a clear psychological component and not a behavioral syndrome, it makes it hard to diagnose accurately if doctors are unfamiliar with the presentation [4]. The diagnostic criteria for AHS are vague and arbitrary. For example, the disinhibited grasp reflex is observed in AHS, however, it is commonly found in focal cerebral injuries or degenerative cerebral disorders. The unilateral arm levitation is seen in cortical basal ganglionic degeneration, that movement is also seen in a progressive form of supranuclear palsy and therefore it is hard to distinguish between these illnesses [5]. When a doctor makes the diagnosis for AHS it involves observation of the physical movements of the patient and an assessment of the frequency and intensity of the random actions being carried out. The motions vary in sudden and disconnected motions or on the more extreme end stealing an individual’s wallet even though it is unintentional [3]. Difficulties in the treatment process are less successful for people with neurodegenerative diseases such as Parkinson’s and Alzheimer’s. The reported treatments in a case study showed a 13-year-old female with right-arm levitation who was prescribed clonazepam and botulinum toxin daily [7]. After two days, there was a 70% reduction in the number of levitations per minute, but she was unable to continue taking clonazepam and maintained botulinum which yielded an 80% improvement in levitations per minute [7]. After medicated treatment, cognitive behavioral therapy, sensory tricks, and visualization strategies are possible treatments. The treatment and diagnosis of AHS do not look at the psychological component of the disease because there are parallels between AHS and the passivity phenomena seen in schizophrenia since both diseases have corpus callosum pathology [2,4]. If a physician were to recognize the characteristic presentation of AHS, it could alleviate the patient’s anxiety and make symptoms easier to deal with. Overall, there are currently no specified routes of treatment for AHS.

**Figure 1:**
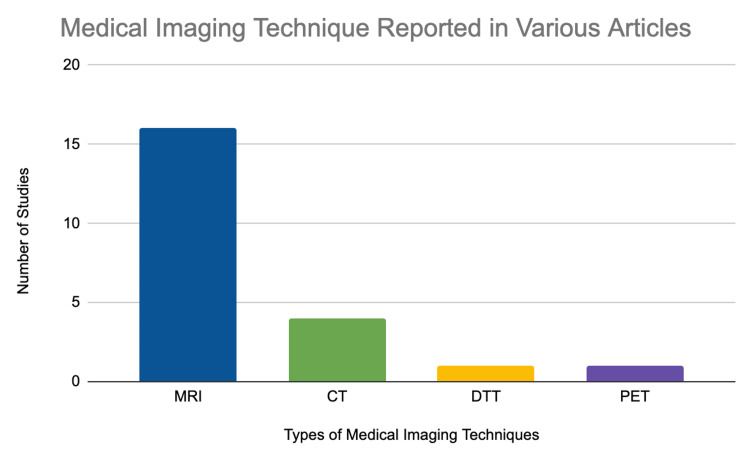
Frequency of Various Medical Imaging Techniques Reported in Different Studies MRI: magnetic resonance imaging; CT: cranial tomography; PET: positron emission tomography; DTT: diffusion tensor tractography

The method diagnosis which is most heavily used is an MRI due to specific brain functionalities that it displays. For example, cranial tomography (CT) scans can be used to generate images of the brain but because they do not use ionizing radiation they are not as detailed as MRI scans [8,9]. Additionally, positron emission tomography (PET) scans utilize radioactive glucose to showcase different parts of the brain which are activated while performing specific tasks but are not useful for generating images of the brain, which are needed to diagnose AHS [10,11]. According to the information presented in Table 1, the most frequently used method for diagnosis of AHS was MRI, specifically 16 out of the 20 studies that were analyzed within the review [7-26]. The second most frequently used method for diagnosis of AHS was CT scan, according to the data presented in Figure 1, four studies within the review reported that CT scans were used to diagnose AHS. This was most likely because it is one of the few techniques that can generate a proper image of the brain. The least commonly used method for diagnosis of AHS was PET scan, within the investigation only one out of the 20 studies reported the use of ​​PET scans to help diagnose AHS [7-26]. This potentially could be due to the fact that PET scans do not successfully generate images of the brain which are necessary for a diagnosis of AHS. Additionally, diffusion tensor tractography (DTT), which is a very specific MRI technique, is another way to diagnose patients that have AHS. This advanced technique measures the rate of water diffusion among cells and helps to create a visual of the internal structures of the human body. Within the review, there was only one study that reported the use of DTT to diagnose AHS [13]. Although DTT is more valuable than MRI, it is not as widely used as it is more expensive, however, as technology improves in the future it could possibly be a mainstream way to diagnose patients with AHS.

**Table 1:** The Method of Diagnosis and the Pre-existing Condition for Alien Hand Syndrome Patients DTT: diffusion tensor tractography; CJD: Creutzfeldt-Jakob Disease; CBS: corticobasal syndrome; CT: cranial tomography; PET: positron emission tomography

Author (year)	Pre-existing condition (s)	Methods of diagnosis	Sample size
Bru et al. (2021) [8]	Stroke	MRI	1
Brugger et al. (2015) [9]	Stroke	MRI, CT	10
Cohen et al. (2016) [10]	Stroke	MRI, CT	77
Brussino et al. (2010) [11]	Stroke	PET scan	4
Iwashita et al. (2005) [12]	Stroke	CT	1
Jang et al. (2013) [13]	Stroke	DTT	1
Kim et al. (2010) [14]	Stroke	MRI	1
Kloesel et al. (2010) [15]	Stroke	MRI	1
Korsakova et al. (2017) [16]	Stroke	N/A	1
Kurne et al. (2008) [17]	Multiple sclerosis	MRI	1
Pack et al. (2002) [18]	Stroke	MRI	1
Pooyania et al. (2011) [19]	Stroke	MRI, CT	1
Rabinstein et al. (2002) [20]	CJD	MRI	1
Ruggerri et al. (2020) [21]	CBS	MRI	12
Sarva et al. (2014) [7]	Stroke	MRI	109
Scepkowski and Cronin-Golomb (2003) [22]	Stroke	MRI	50
Schaefer et al. (2013) [23]	Stroke	MRI	1
Jog and Kumar (2009) [24]	Stroke	MRI	1
Vincent and Hadjikhani (2007) [25]	Migraines	MRI	1
Yuan et al. (2011) [26]	Hypertension, diabetes	MRI	1

AHS has various pre-existing conditions such as stroke, multiple sclerosis, Creutzfeldt-Jakob Disease (CJD), corticobasal syndrome (CBS), migraines, and hypertension. Specifically, according to the data in Table 1, over 15 studies within the review indicate that stroke is the major pre-existing condition for AHS [7-26]. Therefore, it is important to monitor the progression and development of stroke patients to ensure that their condition does not worsen and develop into AHS. Although, only a few studies within the review have mentioned that multiple sclerosis, CJD, CBS, migraines, and hypertension are pre-existing conditions for AHS [17,20-21,25-26]. It is important to still monitor the development of these patients to ensure that they do not develop AHS. Being able to differentiate between the conditions and symptoms of AHS and other neurological conditions is important because AHS is often overlooked and misunderstood for other conditions such as epilepsy. Understanding the pre-existing conditions can reveal a pattern and in the future hopefully a thorough explanation of the biology of the disease which can give helpful information to physicians when treating AHS. Having a close identification of the risk factors and tell-tale signs of the disease can provide physicians with more information about the pre-existing conditions that could potentially explain why a patient with AHS is experiencing a deterioration in their health.

The results presented in this paper can hopefully be utilized by physicians to increase the rate of diagnosis of patients with AHS. Within the review, there were several different pre-existing conditions for AHS that were identified. This information could potentially be utilized to help physicians keep track of patients who are at risk of developing the syndrome and to closely monitor their progression to ensure that their condition does not worsen. The data reported within the article also highlights the importance of using various imaging techniques to help potentially identify different neurological conditions that a patient can have. Therefore, it is quintessential that hospitals and hospital staff invest in maintaining and improving the current standard of imaging technology that can be used to identify harmful conditions within patients before they become issues that cause significant harm. Certain pre-existing conditions for AHS that were identified within the review, such as stroke, are conditions that could potentially be avoided if a patient maintains a healthy standard of living. AHS has gone away after a certain period of time, and by understanding the psychology of the disease physicians can be aware of the possible outcomes of a patient’s neurological condition. This condition is prevalent in 60% of affected individuals facing conditions such as Parkinson’s, palsy, and dementia [2]. Therefore, in order to prevent more patients from developing AHS, it is critical that physicians encourage their patients to exercise and maintain a healthy diet. In order to improve patient outcomes, early identification and close monitoring for prevention of AHS are essential.

Although there were some significant results that were reported within this investigation, there were several limitations to this study. For example, a large volume of case reports were being used in this investigation which did not give enough information and statistics regarding what worked for patients’ treatment of AHS and there have been different approaches to treating the disease. The sample size from the given table is a limitation since most of the findings were case reports, which have varying results regarding the circumstances of a patient’s AHS and so there might have been a small margin of error when compiling data. By having a smaller sample size, it creates a generalization of results to a wider population, which may not be fully representative of the findings and core principles of AHS itself. Since case studies analyze a sample size of one, it is possible to over-analyze the results. The conclusions drawn from other case studies might not be transferable to other settings in the medical field. Therefore, more original research, the symptoms, and causes of AHS should be collected in a clinical and lab setting.

Future investigations of AHS can look to solidify effective treatments of AHS and give a detailed understanding of how the anatomy and physiology of the impacted regions of AHS degenerate. If surgery is done after someone has a stroke, it may be possible to conduct a brain biopsy to eliminate certain diseases and make a diagnosis. A possible future investigation to improve upon current research could be to do a functional MRI (fMRI) experiment that examines the levitation-like movements of a healthy individual and a recent MRI scan of an AHS patient. If a healthy individual could have AHS movements induced, then it could simulate the experience for an individual facing AHS and which regions of the brain are being impacted by the alien movements, possibly looking at the nerves involved in the levitating limbs to target specific behaviors. If individuals participated in the experimental procedure and data was collected while they were conscious and unconscious, it could potentially show that nerve impulses will cause movement and brain activity that could be measured in an fMRI. Understanding the pathology of AHS will help to recognize the neural circuitry that is unique to patients diagnosed with AHS. If it is possible to track and identify the specific functional impairments that lead to the disease and a degenerative state, that might be the next best direction to get individualized supportive care by a physician. Due to the rarity of it being diagnosed in general, having placebo-controlled trials may be difficult in the future.

## Conclusions

AHS is an uncommon condition that causes an individual to lose control of their upper limbs resulting in their movement being limited. Additionally, this disease does occur without a severe pre-existing condition. Based upon the results of this study, it was discovered that stroke is the most common pre-existing condition among individuals who had AHS. The most frequently used method of diagnosis found in the study was MRI, this was most likely due to the fact that it was the best at displaying the brain functionalities of various patients. Potentially future investigations will determine more information regarding the causes of AHS as well as develop better methods of diagnosing patients with AHS. Ultimately, physicians should closely monitor their AHS patients primarily because it could reveal more information about how treatment of AHS could be improved in the future.
